# Prevalence and Clinical Factors Associated with Self-reported Smell and Taste Disorders in Older Adults Hospitalized with COVID-19

**DOI:** 10.1055/s-0045-1801854

**Published:** 2025-07-29

**Authors:** Letícia de Carvalho Palhano Travassos, Hemílio Fernandes Campos Coelho, Assel Muratovna Shigayeva Ferreira, Leandro Pernambuco

**Affiliations:** 1 Departament of Statistics, Centro de Ciências Exatas e da Natureza, Universidade Federal da Paraíba, João Pessoa, PB, Brazil; 2 Department of Speech Therapy, Centro de Ciências da Saúde, Universidade Federal de Pernambuco, Recife, PE, Brazil

**Keywords:** elderly, ageusia, anosmia, hospitalization, COVID-19, logistic models

## Abstract

**Introduction:**

Complaints of smell and taste disorders are present in people with coronavirus disease 2019 (COVID-19), and they particularly impact older adults in their daily activities and quality of life. Understanding these disorders in this specific population is crucial due to the heightened susceptibility to decreased general health.

**Objective:**

To assess the prevalence and the factors associated with self-reported smell and taste disorders in older adults hospitalized with COVID-19.

**Methods:**

The present documentary and retrospective study used a dataset from the Paraíba State Department of Health based on individual record sheets of hospitalized people with severe acute respiratory syndrome (SARS), collected through a national form routinely applied in Brazilian hospitals. The complaints of smell and taste disorders were the dependent variables. The independent variables included the clinical outcomes and comorbidities. Data analysis involved descriptive statistics, the Fisher's exact test, and binary logistic regression. The confidence interval was 95%.

**Results:**

The sample comprised 5,014 older adults with a mean age of 74.50 ± 9.35 years, of both biological sexes, and most of them were admitted to the Intensive Care Unit (ICU), required non-invasive respiratory support, and experienced death. The prevalence of self-reported smell and taste disorders was 7.8% (95%CI = 7.2–8.8%) and 6.4% (95%CI = 5.6–7.1%) respectively. Smell disorders were less frequent among subjects admitted to the ICU and those who died, while taste disorders correlated with clinical outcomes such as fever, cough, sore throat, diarrhea, and comorbidities such as chronic neurological disease.

**Conclusion:**

Self-reported smell and taste disorders are present in almost 10% of older adults hospitalized with COVID-19, and they are associated with clinical outcomes and commorbities.

## Introduction

In January 2020, Chinese researchers identified a new type of coronavirus (severe acute respiratory syndrome coronavirus 2, SARS-CoV-2), belonging to the genus β, as the etiological agent of SARS, called *coronavirus disease 2019* (COVID-19). 1 The group most vulnerable to the virus is men older than 50 years who have comorbidities such as diabetes, hypertension, cardiovascular diseases, and cerebrovascular diseases. Its highest mortality is among the elderly. 2

Older adults present weakened physiological functioning of vital organs, including the respiratory system, and reduced immunity, which increases the likelihood of developing chronic diseases and of the establishment of viral infections. 3 These aspects, together with immobility in bed, leave these individuals more vulnerable to complications, making the hospitalization process complex because it depends on the influence of multiple factors of the hospital environment itself. 4

The symptoms of COVID-19 may range from mild to severe. The most common initial symptoms are cough, fever, fatigue, headache, myalgia, diarrhea, and smell and taste disorders. 5 Smell and taste are essential sensory functions; therefore, olfactory and gustatory dysfunctions have an important impact on the patients' quality of life, affecting both the ability to experience rewards related to smell and taste and the ability to detect odors, flavors, and potentially harmful substances. 6

Regarding smell and taste disorders, a study 7 conducted with 2,581 adult individuals assessed the prevalence of anosmia in mild to critical cases of COVID-19, and the authors found that the prevalence of self-reported anosmia was of 85.9% in mild cases, of 4.5% in moderate cases, and of 6.9% in severe-to-critical cases. Studies 8 have related the loss of smell and taste to a milder course of the disease and have reported a greater occurrence in young patients. 8 However, no studies have explored the prevalence and clinical factors associated with the loss of smell and taste only among the elderly.

Thus, the aim of the current study was to analyze the prevalence and clinical factors associated with self-reported smell and taste disorders in older adults hospitalized with COVID-19.

## Methods

The present is a documentary and retrospective study submitted to and approved by the0 institutional Ethics in Research Committee under protocol number 4.174.541.

The study used a dataset from the Department of Health of the state of Paraíba, Northeastern Brazil regarding the mandatory notifications of individual record sheets of patients hospitalized with SARS. The data was collected by trained healthcare workers in the hospital using a standardized form prepared by the Brazilian Ministry of Health in partnership with the Brazilian Health Regulatory Agency (Agência Nacional de Vigilância Sanitária, ANVISA, in Portuguese) that is used nationwide as a routine.

The form is divided into general information and clinical and epidemiological outcomes. For the current study, data on smell and taste disorders complaints was treated as the dependent variable. The independent variables were data on clinical outcomes divided into signs and symptoms (fever, cough, sore throat, dyspnea, respiratory distress, oxygen saturation < 95%, diarrhea, vomiting, abdominal pain, and/or fatigue) and comorbidities (cardiovascular disease, hematological disease, liver disease, asthma, diabetes, neurological disease, other lung diseases, immunodeficiency or immunosuppression, kidney disease, and/or obesity). Information on admission to the Intensive Care Unit (ICU), use of respiratory support, and case evolution (recovery/death) was also collected.

**Fig. 1:**
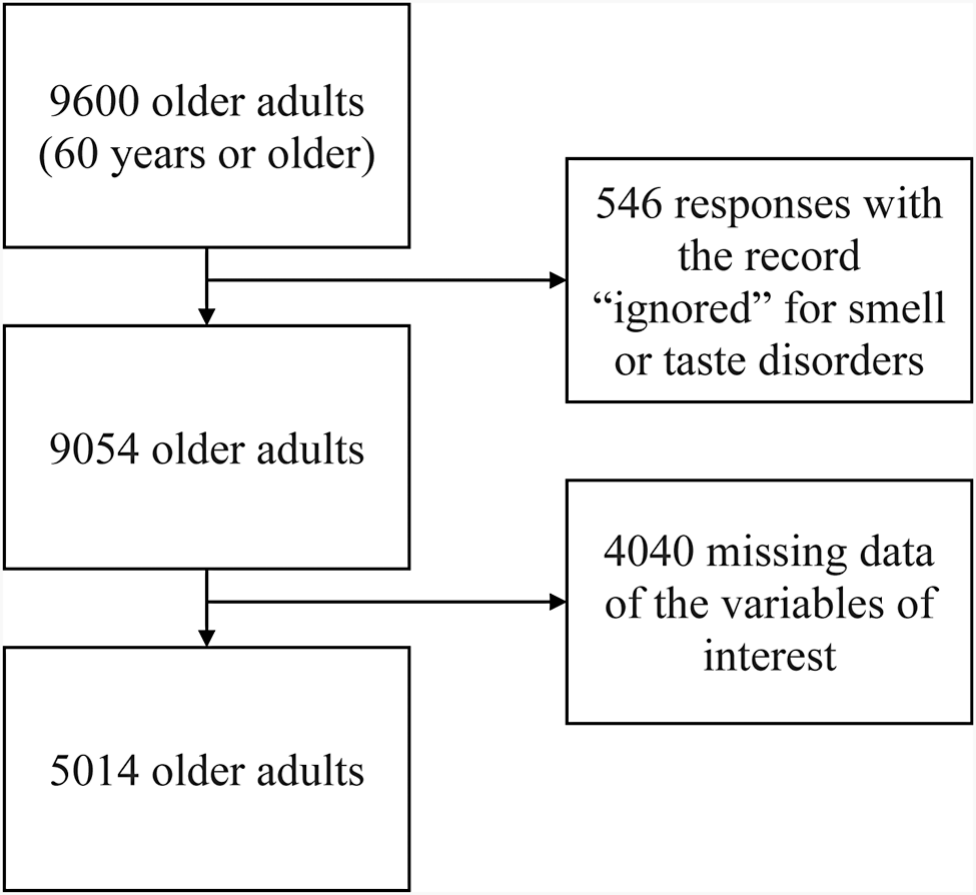
Flowchart of the selection of the sample of the present study.

The inclusion criteria were individuals of both biological sexes, aged 60 years or older, diagnosed with SARS, with confirmed diagnosis of COVID-19, and hospitalized in the network of the Brazilian Unified Health System (Sistema Único de Saúde, SUS, in Portuguese) between January and December 2020. The exclusion criteria were individuals with “ignored” records or missing data for smell or taste disorders, or the other variables that were of interest in the study, as shown in Fig. 1 .

The data were analyzed using PSPP software (free; https://www.gnu.org/software/pspp/ ). The descriptive analysis of the quantitative variables used measures of central tendency (mean and median) and dispersion (standard deviation). The categorical variables were expressed as numbers and percentages. The associations involving smell and taste disorders and the independent variables were assessed through Fisher's exact test, and the prevalence ratio was the measure of association. The confidence interval was 95% (95%CI). The variables with values < 0.2 in the Fisher's exact test were added to the binary logistic regression. The adequacy of the logistic regression model was assessed using the Hosmer–Lemeshow test.

## Results

The final sample consisted of 5,014 individuals aged 60 years or older (mean age: 74.50 ± 9.35 years) of both biological sexes (male patients: *n* = 2,543; 50.7%). Among these individuals, 2,577 (51.4%) were admitted to the ICU, 2,880 (57.4%) required noninvasive ventilatory support, 1,698 (33.9%) required invasive ventilatory support, and 436 (8.7%) did not require ventilatory support. A total of 2,794 (55.7%) patients died.

**Fig. 2:**
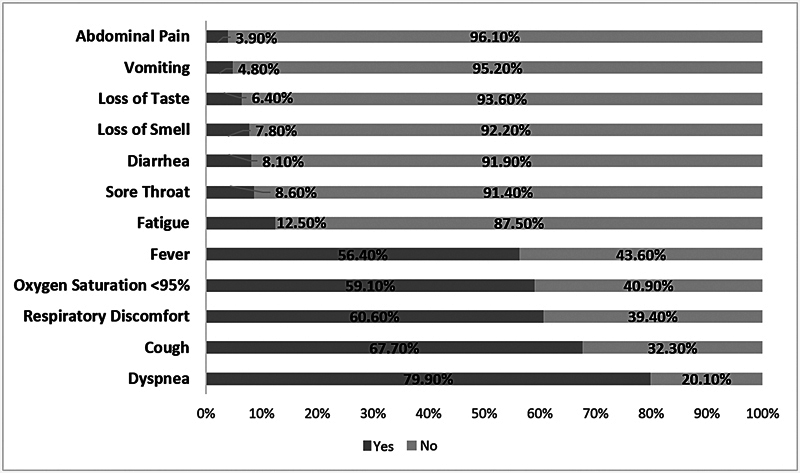
Percentage distribution of the clinical outcomes of older adults hospitalized with coronavirus disease 2019 (COVID-19) in the state of Paraíba, Northeastern Brazil, in 2020 ( *n* = 5,014).

**Fig. 3:**
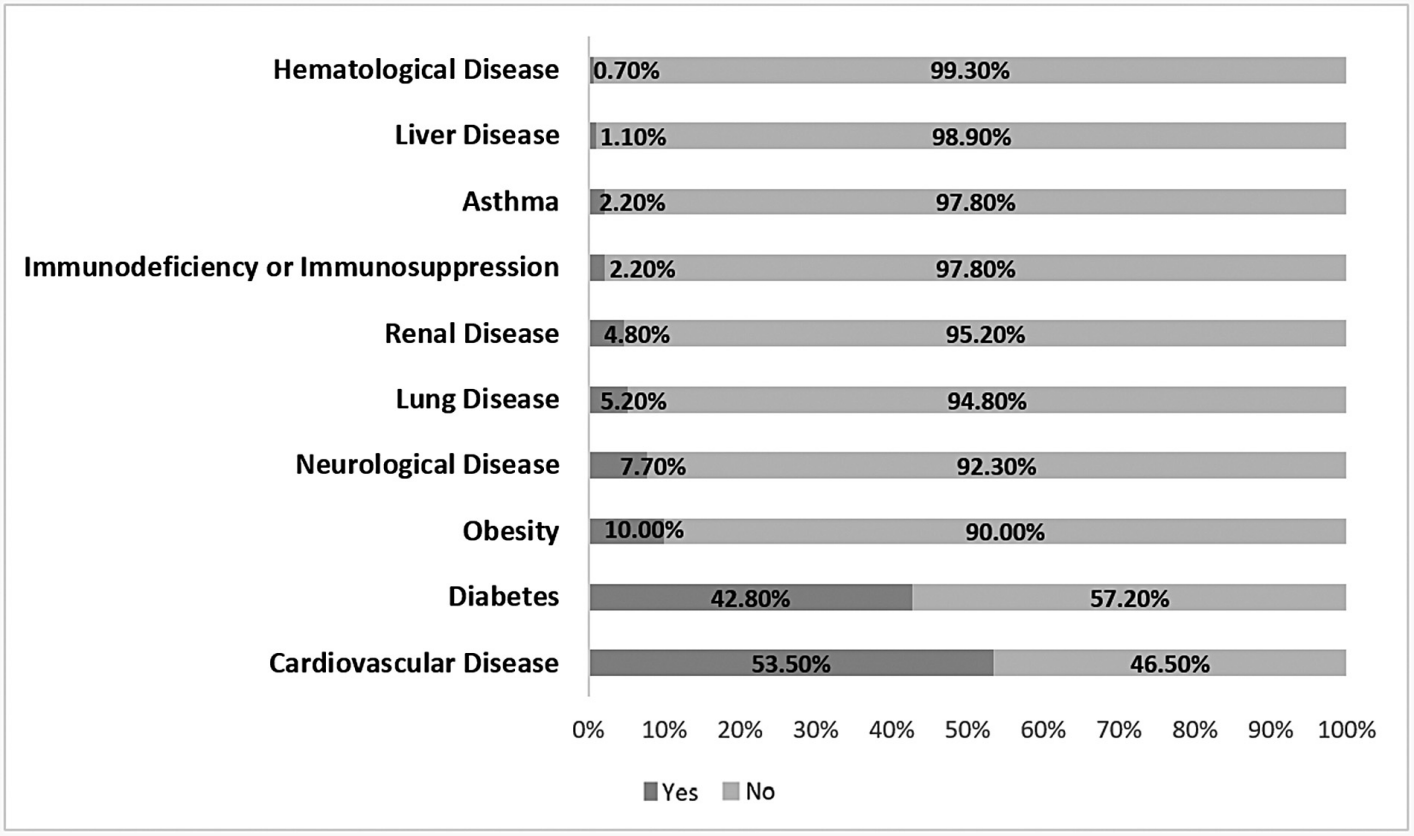
Percentage distribution of comorbidities presented by older adults hospitalized with COVID-19 in the state of Paraíba, Northeastern Brazil, in 2020.

The prevalence of self-reported smell and taste disorders was 7.8% (95%CI = 7.2–8.8%) and 6.4% (95% CI = 5.6–7.1%) respectively. As seen in Fig. 2 , the most frequent clinical outcomes were dyspnea (79.9%), cough (67.7%), respiratory distress (60.60%), oxygen saturation < 95% (59.1%), and fever (56.4%). The most frequent comorbidities were cardiovascular disease (53.5%) and diabetes (42.8%) ( Fig. 3 ).

Table 1 shows the associations involving the clinical outcomes and smell and taste disorders. The frequency of smell disorders was higher among patients who presented fever, cough, sore throat, respiratory distress, diarrhea, vomiting, abdominal pain, fatigue, or asthma, and it was significantly lower in subjects admitted to the ICU and in those who died ( Table 1 ).The prevalence of taste disorders was higher in patients who presented fever, cough, sore throat, diarrhea, vomiting, abdominal pain, or fatigue, while it was lower in those with chronic neurological diseases ( Table 1 ).

**Table 1:** Distribution of self-reported smell and taste disorders according to clinical outcomes and comorbidities in older adults hospitalized with COVID-19 in the state of Paraíba, Northeastern Brazil ( *n* = 5,014)

**Variables**	**Self-reported smell disorders**				**Self-reported taste disorders**			
	**No** **(n = 4,623):**	**Yes** **(n = 391):**	***p*** *	**PR (95%CI)**	**No** **(n = 4,693):**	**Yes** **(n = 321):**	***p*** *	**PR (95%CI)**
	**n (%)**	**n (%)**			**n (%)**	**n (%)**		
**Gender**								
Male	2,346 (92.3)	197 (7.7)	0.916	0.99 (0.82- 1.2)	2,304 (93.2)	167 (6.8)	0.327	1.12 (0.91–1.38)
Female	2,277 (92.1)	194 (7.9)			2,389 (93.9)	154 (6.1)		
**Fever**								
No	2,053 (94.0)	131 (6.0)	0.000*	0.65 (0.53–0.8)	2,077 (95.1)	107 (4.9)	0.000*	0.65 (0.52–0.81)
Yes	2,570 (90.8)	260 (9.2)			2,616 (89.3)	214 (7.6)		
**Cough**								
No	1,541 (95.2)	77 (4.8)	0.000*	0.51 (0.4–0.65)	1,555 (96.1)	63 (3.9)	0.000*	0.51 (0.39–0.67)
Yes	3,082 (90.8)	314 (9.2)			3,138 (92.4)	258 (7.6)		
**Sore throat**								
No	4,256 (92.9)	326 (7.1)	0.000*	0.47 (0.37–0.6)	4,312 (94.1)	270 (5.9)	0.000*	0.5 (0.38–0.66)
Yes	367 (85.0)	65 (15.0)			381 (88.2)	51 (11.8)		
**Dyspnea**								
No	922 (91.4)	87 (8.6)	0.152	1.14 (0.91–1.43)	944 (93.6)	65 (6.4)	0.501	1.01 (0.78–1.31)
Yes	3,701 (92.4)	304 (7.6)			3,749 (93.6)	256 (6.4)		
**Respiratory discomfort**								
No	1,854 (93.9)	121 (6.1)	0.000*	0.69 (0.56–1.85)	1,865 (94.4)	110 (5.6)	0.029	0.8 (0.64–1.0)
Yes	2,769 (91.1)	270 (8.9)			2,828 (93.1)	211 (6.9)		
**Oxygen saturation < 95%**								
No	1,901 (92.7)	149 (7.3)	0.133	0.89 (0.73–1.08)	1,929 (94.1)	121 (5.9)	0.126	0.87 (0.7–1.08)
Yes	2,722 (91.8)	242 (8.2)			2,764 (93.3)	200 (6.7)		
**Diarrhea**								
No	4,278 (92.8)	331 (7.2)	0.000*	0.48 (0.37–0.62)	4,338 (94.1)	271 (5.9)	0.000*	0.48 (0.36–0.64)
Yes	345 (85.2)	60 (14.8)			355 (87.7)	50 (12.3)		
**Vomiting**								
No	4,422 (92.2)	352 (7.4)	0.000*	0.45 (0.33–0.61)	4,481 (93.9)	293 (6.1)	0.001*	0.53 (0.37–0.76)
Yes	201 (83.8)	39 (16.3)			212 (88.3)	28 (11.7)		
**Abdominal pain**								
No	4,478 (93.0)	339 (7.0)	0.000*	0.27 (0.21–0.35)	4,542 (94.3)	275 (5.7)	0.000*	0.24 (0.18–0.32)
Yes	145 (73.6)	52 (26.4)			151 (76.6)	46 (23.4)		
**Fatigue**								
No	4,105 (93.6)	280 (6.4)	0.000*	0.36 (0.29–0.44)	4,166 (95.0)	219 (5.0)	0.000*	0.31 (0.25–0.39)
Yes	518 (82.4)	111 (17.6)			527 (83.8)	102 (16.2)		
**Chronic cardiovascular disease**								
No	2,168 (93.0)	163 (7.0)	0.027	0.82 (0.68–0.99)	2,192 (94.0)	139 (6.0)	0.130	0.88 (0.71–1.09)
Yes	2,455 (91.5)	228 (8.5)			2,501 (93.2)	182 (6.8)		
**Chronic hematological disease**								
No	4,590 (92.2)	387 (7.8)	0.326	0.72 (0.28–1.83)	4,659 (93.6)	318 (6.4)	0.426	0.79 (0.27–2.35)
Yes	33 (89.2)	4 (10.8)			34 (91.9)	3 (8.1)		
**Chronic liver disease**								
No	4,571 (92.2)	386 (7.8)	0.461	0.89 (0.38–2.07)	4,640 (93.6)	317 (6.4)	0.500	0.91 (0.35–2.36)
Yes	52 (91.2)	5 (8.8)			53 (93.0)	4 (7.0)		
**Asthma**								
No	4,530 (92.3)	376 (7.7)	0.019*	0.55 (0.34–0.89)	4,595 (93.7)	311 (6.3)	0.152	0.68 (0.37–1.24)
Yes	93 (86.1)	15 (13.9)			98 (90.7)	10 (9.3)		
**Diabetes**								
No	2,663 (92.9)	204 (7.1)	0.021	0.82 (0.68–0.99)	2,698 (94.1)	169 (5.9)	0.051	0.83 (0.67–1.03)
Yes	1,960 (91.3)	187 (8.7)			1,995 (92.9)	152 (7.1)		
**Chronic neurological disease**								
No	4,258 (92.0)	369 (8.0)	0.060	1.4 (0.92–2.13)	4,318 (93.3)	309 (6.7)	0.002*	2.15 (1.22–3.79)
Yes	365 (94.3)	22 (5.7)			375 (96.9)	12 (3.1)		
**Chronic lung disease**								
No	4,383 (92.2)	370 (7.8)	0.474	0.97 (0.64–1.48)	4,449 (93.6)	304 (6.4)	0.508	0.98 (0.61–1.57)
Yes	240 (92.0)	21 (8.0)			244 (93.5)	17 (6.5)		
**Immunodeficiency or immunosuppression**								
No	4,527 (92.3)	378 (7.7)	0.080	0.65 (0.39–1.09)	4,592 (93.6)	313 (6.4)	0.398	0.87 (0.44–1.71)
Yes	96 (88.1)	13 (11.9)			101 (92.7)	8 (7.3)		
**Chronic kidney disease**								
No	4,394 (92.1)	379 (7.9)	0.054	1.59 (0.91–2.78)	4,462 (93.5)	311 (6.5)	0.086	1.57 (0.85–2.91)
Yes	229 (95.0)	12 (5.0)			231 (95.9)	10 (4.1)		
**Obesity**								
No	4,170 (92.4)	343 (7.6)	0.072	0.79 (0.59–1.05)	4,233 (93.8)	280 (6.2)	0.056	0.76 (0.55–1.04)
Yes	453 (90.4)	48 (9.6)			460 (91.8)	41(8.2)		
**ICU admission**								
No	2,221 (91.1)	216 (8.9)	0.004*	1.31 (1.08–1.59)	2,265 (92.9)	172 (7.1)	0.037*	1.22 (0.99–1.51)
Yes	2,402 (93.2)	175 (6.8)			2,428 (94.2)	149 (5.8)		
**Ventilatory support**								
Yes, invasive	1,575 (92.8)	123 (7.2)	0.577	0.9 (0.73–1.11)	1,598 (94.1)	100 (5.9)	0.452	0.9 (0.71–1.14)
Yes, non-invasive	2,647 (91.9)	233 (8.1)			2,691 (93.4)	189 (6.6)		
No	401 (92.0)	35 (8.0)			404 (92.7)	32 (7.3)		
**Case evolution**								
Recovery	2,023 (91.1)	197 (8.9)	0.013*	1.28 (1.06–1.55)	2,059 (92.7)	161 (7.3)	0.032	1.27 (1.03–1.57)
Death	2,600 (93.1)	194 (6.9)			2,634 (94.3)	160 (5.7)		
**Variants**								
B. 1.1.28 or B.1.1.33 (VOC)	4,069 (92.3)	338 (7.7)	0.374	0.88 (0.67–1.16)	4,137 (93.9)	270 (6.1)	0.041*	0.73 (0.55–0.97)
P.1 or P.2 (VOI)	554 (91.3)	53 (8.7)			556 (91.6)	51 (8.4)		

**Abbreviations:** 95%CI, 95% confidence Interval; COVID-19, coronavirus disease 2019; ICU, intensive care unit; PR, prevalence ratio; VOC, variant of concern; VOI, variant of interest.

**Note:** * *p* < 0.05 (Fisher's exact test).

The results of the binary logistic regression model for smell and taste disorders can be observed in Table 2 and Table 3 respectively. Both models indicated a good fit, as the result was > 0.05 in the Hosmer-Lemeshow test (0.440 for the smell model and 0.357 for the taste model).

**Table 2:** Results of the binary logistic regression model adjusted for smell disorders in older adults hospitalized with COVID-19 in the state of Paraíba, Northeastern Brazil, 2020 ( *n* = 5,014)

**Variable**	**Coefficient estimate**	***p*** **-value**	**OR**	**95%CI**		**PR**	**95%CI**		**Classification**
				**LL**	**UL**		**LL**	**UL**	
Fever (yes)	0.253	0.032*	1.288	1.022	1.623	1.247	1.018	1.529	Risk factor
Cough (yes)	0.509	0.000*	1.663	1.266	2.184	1.568	1.227	2.004	Risk factor
Sore throat (yes)	0.420	0.008*	1.522	1.115	2.078	1.435	1.101	1.871	Risk factor
Dyspnea (yes)	−0.266	0.051	0.767	0.587	1.001	0.795	0.633	0.998	Protective factor
Respiratory discomfort (yes)	0.383	0.002*	1.467	1.157	1.861	1.400	1.134	1.727	Risk factor
Diarrhea (yes)	0.481	0.003*	1.617	1.172	2.232	1.510	1.150	1.982	Risk factor
Vomiting (yes)	0.440	0.033*	1.553	1.036	2.328	1.457	1.036	2.049	Risk factor
Abdominal pain (yes)	1.123	0.000*	3.075	2.111	4.478	2.563	1.905	3.449	Risk factor
Fatigue (yes)	0.801	0.000*	2.228	1.723	2.881	2.002	1.607	2.494	Risk factor
Asthma (yes)	0.633	0.033*	1.883	1.052	3.371	1.703	1.062	2.731	Risk factor
Diabetes (yes)	0.210	0.054	1.234	0.996	1.528	1.200	0.996	1.444	Risk factor
Renal disease (yes)	−0.561	0.073	0.570	0.309	1.053	0.607	0.348	1.058	Protective factor
ICU admission (yes)	−0.305	0.006*	0.737	0.593	0.916	0.767	0.634	0.927	Protective factor
Constant	−3.336	0.000	−	−	−				−

**Abbreviations:** 95%CI, 95% confidence Interval; COVID-19, coronavirus disease 2019; ICU, intensive care unit; LL, lower limit; OR, odds ratio; PR, prevalence ratio; UL, upper limit.

**Note:** * *p* < 0.05 (Fisher's exact test).

**Table 3:** Results of the logistic regression model adjusted for taste disorders in older adults hospitalized with COVID-19 in the state of Paraíba, Northeastern Brazil, 2020 ( *n* = 5,014)

**Variable**	**Coefficient estimate**	***p*** **-value**	**OR**	**95%CI**		**PR**	**95%CI**		**Classification**
				**LL**	**UL**		**LL**	**UL**	
Fever (yes)	0.296	0.021*	1.345	1.045	1.731	1.307	1.039	1.644	Risk factor
Cough (yes)	0.565	0.000*	1.759	1.307	2.366	1.675	1.273	2.205	Risk factor
Sore throat (yes)	0.426	0.012*	1.531	1.096	2.139	1.462	1.089	1.963	Risk factor
Respiratory discomfort (yes)	0.229	0.070	1.257	0.981	1.611	1.229	0.982	1.539	Risk factor
Diarrhea (yes)	0.555	0.001*	1.742	1.243	2.442	1.638	1.219	2.201	Risk factor
Abdominal pain (yes)	1.478	0.000*	4.385	3.032	6.342	3.557	2.661	4.757	Risk factor
Diabetes (yes)	0.202	0.087	1.224	0.971	1.544	1.199	0.974	1.477	Risk factor
Neurological disease (yes)	−0.697	0.021*	0.498	0.275	0.900	0.525	0.301	0.917	Protective factor
Case evolution (recovery)	0.268	0.025*	1.308	1.033	1.655	1.273	1.030	1.573	Risk factor
Variant: B.1.1.28 or B.1.1.33 (VOC)	−0.427	0.009*	0.652	0.474	0.899	0.684	0.517	0.905	Protective factor
Constant	−3.456	0.000	0.032	−	−				−

**Abbreviations:** 95%CI, 95% confidence interval; COVID-19, coronavirus disease 2019; LL, lower limit; OR, odds ratio; PR, prevalence ratio; UL, upper limit; VOC, variant of concern.

**Note:** * *p* < 0.05 (Fisher's exact test).

## Discussion

The current study examined the prevalence and factors associated with self-reported smell and taste disorders in older adults hospitalized with COVID-19 in the state of Paraíba, Northeastern Brazil. The prevalence rates for these disorders in the studied population were close to 10%, and they were associated with clinical outcomes and comorbidities.

The findings of the present study corroborate those of the literature, which shows that the prevalence of smell and taste disorders was observed less frequently in geriatric patients, which is the population included in the current study. 9 The exact physiopathology of smell and taste disorders in COVID-19 remains controversial, 10 and their prevalence varies greatly among populations, ranging from 3.2% to 98.3% for smell disorders, and from 5.6% to 62.7% for taste disorders. 11

Such differences among populations can be due to biological sex, age, intrinsic human host features, the degree of severity of COVID-19, the sample size, and the diagnostic method employed. 12 Studies employing self-reported symptoms of smell and taste has identified a lower prevalence than studies utilizing some instrumental assessment. 13

Additionally, it has been shown that the older the patient, the lower the incidence of these dysfunctions. 11 It has been hypothesized that many older individuals already perceived sensory modifications as a normal part of aging, with no association with COVID-19 infection. This could explain the lower prevalence of smell and taste disorders in studies relying on self-reports of older adults compared with younger adults 26 . Despite these alterations being underreported in the elderly, they can have significant impacts on the quality of life of this population, including negative emotional impacts, feelings of isolation, impaired relationships, impaired daily functioning, and impacts on physical health. 14

Regarding the clinical outcomes associated with self-reported smell disorders, the binary logistic regression analysis revealed that fever, cough, sore throat, respiratory discomfort, diarrhea, vomiting, abdominal pain, fatigue, and asthma increase the likelihood of an individual experiencing smell disorders, while ICU admission reduces this likelihood. For self-reported taste disorders, there was a higher prevalence in the presence of fever, cough, sore throat, diarrhea, abdominal pain, and recovery. Meanwhile, the presence of neurological disease and the B.1.1.28 or B.1.1.33 variants, which were classified *variants of concern* (VOCs) by the World Health Organization (WHO) based on several factors, including transmissibility, virulence, phenotypic changes, and propagation, 15 resulted in a lower chance of developing these symptoms.

The literature 16 17 has shown that there are no significant associations involving general COVID-19 symptoms (fever, cough, sore throat, myalgia, headaches, diarrhea, rhinorrhea) and olfactory or gustatory dysfunctions. However, a study 16 showed that 76.4% of COVID-19patients who complained of smell disorders and 78% of those who complained of taste disorders also had symptoms of fever; in addition, cough was present in 53.2% of the subjects with smell disorders and in 53.9% of those with taste disorders.

In another study, linear regression revealed a significant correlation between the severity of olfactory and gustatory dysfunction and fever. It is believed that, at the beginning of COVID-19, smell and taste disorders are not associated with other symptoms. However, the maintenance of these symptoms may indicate that the virus is still present in the upper respiratory tract, which may prolong certain symptoms, especially fever. 18

The other symptoms that increase the likelihood of the subjects developing smell and taste disorders are considered initial and mild in COVID-19 infection. This is justified, as smell and taste disorders typically occur early in the disease (within five days) and are associated with a milder clinical picture. 19 More severe symptoms, such as dyspnea, showed no association with the development of smell and taste disorders, and they are often linked to severe cases and fatal outcomes. 20

Some variables representing more severe outcomes showed associations with lower frequencies of self-reported smell and taste disorders. The proportion of patients with self-reported smell disorders was lower in cases of ICU admission, and the presence of self-reported taste disorders was lower in individuals who died. There is evidence that the presence of olfactory dysfunction is associated with a milder clinical course and, in particular, a decreased risk of pneumonia, lower levels of inflammatory markers, reduced need for hospitalization, decreased need for oxygen therapy, lower ICU admission rates, decreased intubation requirements, and reduced mortality. The presence of gustatory dysfunction was associated with a decreased risk of developing pneumonia and a reduced need for hospitalization. 21 22

Regarding comorbidities, in the current study, individuals with neurological diseases presented a lower frequency of self-reported smell and taste disorders. One of the primary etiologies of taste disorders is neurological diseases, 23 which contradicts the findings of the present study study. One hypothesis is that these patients already had taste disorders before COVID-19; therefore, they did not notice significant differences to report the alteration, or they may have been unable to clearly express what they were feeling.

In the present study, we also found an association between asthma and self-reported smell disorders. In the logistic regression model, asthma increased the chances of an individual experiencing loss of smell. This finding was also reported in a systematic review of adult and elderly patients with COVID-19. 24 In another study, 25 a high prevalence of olfactory dysfunction was observed in patients with upper airway diseases, including asthma, and it was associated with conductive and neurosensory olfactory dysfunctions, including mechanical obstruction of odor transmission in the olfactory cleft due to mucosal inflammation.

Another important finding relates to the variant to which the patient has been exposed. In the current study, the results indicated a lower risk of developing loss of taste when patients were exposed to the B.1.1.28 or B.1.1.33 variants (VOCs). These variants were considered more transmissible and lethal when compared with previously dominant strains, which may justify the fact that being exposed to a variant that leads to a more severe disease is a protective factor for taste disorders, as this symptom occurs more frequently in mild cases of the disease. 25

The present study has certain limitations: the cross-sectional design does not enable us to establish cause-and-effect relationships; the data were self-reported and may have been influenced by memory and response biases; the sample could have been even larger had there been fewer missing data on the variables of interest (even so, the sample was quite large); and since the database came from a routine that was already established at that time, information on smell and taste disorders prior to COVID-19 was not asked, which did not enable us to add it as an exclusion criteria.

The results of the current study contribute to our understanding of the magnitude of the smell and taste disorders in hospitalized older people, especially considering its large sample of patients with COVID-19. The data may help improve the tracking of these symptoms based on knowledge about the associated factors and contribute to more assertive decision-making and management in this population. Future studies may benefit from the longitudinal follow-up of these patients and the use of clinical and/or instrumental assessments to complement the diagnostic investigation of smell and taste disorders and their associated factors.

## Conclusion

In older adults hospitalized with COVID-19, the prevalence of self-reported smell and taste disorders was close to 10%. The clinical outcomes that increased the likelihood of experiencing smell disorders included fever, cough, sore throat, respiratory discomfort, diarrhea, vomiting, abdominal pain, fatigue, and asthma, while ICU admission reduced this likelihood. There was a higher chance of experiencing taste in the presence of fever, cough, sore throat, diarrhea, abdominal pain, and recovery. However, the presence of neurological disease and the B.1.1.28 or B.1.1.33 variants (VOCs) resulted in a lower chance of self-reporting taste disorders.
